# Revision of the lacewing genus *Laccosmylus* with two new species from the Middle Jurassic of China (Insecta, Neuroptera, Saucrosmylidae)

**DOI:** 10.3897/zookeys.790.28286

**Published:** 2018-10-15

**Authors:** Hui Fang, Dong Ren, Jiaxi Liu, Yongjie Wang

**Affiliations:** 1 College of Life Sciences, Capital Normal University, Beijing 100048, China Capital Normal University Beijing China

**Keywords:** Daohugou, Inner Mongolia, Jiulongshan Formation, Mesozoic, Ningcheng

## Abstract

The genus *Laccosmylus* Ren & Yin, 2003 belonging to Saucrosmylidae was erected by using a single hind wing only. Based on new fossil material and re-examination of the type specimen, the diagnosis of the genus is emended with supplementary forewing characters, reported for the first time. In addition, two new species *Laccosmyluscicatricatus***sp. n.** and *Laccosmyluslatizonus***sp. n.** are described.

## Introduction

The family Saucrosmylidae is an enigmatic lineage of Neuroptera, characterised by the typically large body size, extensively expanded RA-RP area and complicated venation (Ren and Yin 2003, Fang et al. 2015). It was originally considered to be related to Osmylidae (Ren and Yin 2003, Winterton et al. 2017). However, a phylogenetic analysis conducted by Yang et al. assigned it within Myrmeleontiformia (Yang et al. 2012). Engel et al. (2018) pointed out the family was possibly a stem group of Osmylidae or Osmyloidae. Therefore, the phylogenetic position of the family is still not resolved.

Notably, the saucrosmylids are restricted to two Middle Jurassic localities, i.e., the Jiulongshan Formation of China and the Kubekovo locality of Russia. To date, eight genera with nine species have been formally described (Ren and Yin 2003, Wang et al. 2010, Liu et al. 2013, Liu et al. 2014, Fang et al. 2015, Khramov 2017). Despite the relatively low specific diversity, saucrosmylids show distinctive morphological diversity, especially various wing markings, e.g., *Saucrosmylus* with transverse bands, *Bellinympha* with distinct pinnate leaf-like markings, which were possibly related to their specialised adaptations to the Mesozoic environments (Wang et al. 2010). Among the known saucrosmylids, the genus *Laccosmylus* Ren & Yin, 2003 was erected based on a single hind wing (Ren and Yin 2003), with characteristic irregular dark and light patches.

This type of taxonomic treatment, i.e., establishing a new taxon based on a single hind wing, is common among Saucrosmylidae and other large neuropterans (Fang et al. 2015). For example, *Rudiosmylus* Ren & Yin, 2003, *Laccosmylus* Ren & Yin, 2003 and *Daohugosmylus* Liu et al., 2014 (Ren and Yin 2003, Liu et al. 2014) were established based on the hind wing only. Although single hind wing could provide some informative characters, the absence of forewing of the genus might result in incomplete information for the overall comparison with other saucrosmylid genera. Herein, we provide an emended diagnosis of *Laccosmylus* with additional forewing characters, re-describe the type species *Laccosmyluscalophlebius* Ren & Yin, 2003 based on the re-examination of the type specimen and additional material, and describe two new species *Laccosmyluscicatricatus* sp. n. and *Laccosmyluslatizonus* sp. n.

## Materials and methods

All the specimens reported here were collected from the Middle Jurassic Jiulongshan Formation at Daohugou Village, Ningcheng County, Inner Mongolia, northeastern China (Ren et al. 2010; Gu et al. 2012; Wang et al. 2012; Meng et al. 2017). All photographs were taken using Canon 70D digital camera, and modified by Photoshop CC. The line drawings were prepared on photographs using the image-editing software CorelDRAW X7. The venation terminology follows Kukalová-Peck and Lawrence (2004) as interpreted by Yang et al. (2012, 2014) and Breitkreuz et al. (2017). Abbreviations of wing veins are as follows (those used in the traditional terminology in parentheses):

**ScP** (= Sc, subcosta) subcostal posterior;

**R** radius;

**RA** (= R1, first branch of radius) radius anterior;

**RP** (= Rs, radial sector) radius posterior;

**RP1** (= Rs1) proximal-most branch of RP;

**RP2** (= Rs2) branch of RP distal to RP1;

**M** media;

**MA** media anterior;

**MP** media posterior;

**Cu** cubitus;

**CuA** cubitus anterior;

**CuP** cubitus posterior;

**AA1-AA3** (= 1A-3A) first to third anal veins.

## Systematic palaeontology

### Class Insecta Linnaeus, 1758

#### Order Neuroptera Linnaeus, 1758

##### Family Saucrosmylidae Ren & Yin, 2003

###### Genus Laccosmylus

Taxon classificationAnimaliaNeuropteraSaucrosmylidae

Ren & Yin, 2003

####### Type species.

*Laccosmyluscalophlebius* Ren & Yin, 2003.

####### Species included.

*Laccosmyluscalophlebius* Ren & Yin, 2003; *Laccosmyluscicatricatus* sp. n.; *Laccosmyluslatizonus* sp. n.

####### Emended diagnosis.

Large body size, body length more than 35 mm, forewing length approx. 60–80 mm, hind wing length approx. 55–76 mm. Forewing elongated, with undulant outer margin. Hind wing broader and shorter than forewing, with slightly undulant outer margin. Forewing and hind wing with similar venation, i.e., presence of 6–7 rows of smaller veinlets between costal veinlets; RA-RP area expanded, with 4 to 7 rows of irregular cells; RP sharply bent towards RA anteriorly, forming an angle of approx. 45°; space between other longitudinal veins producing 1 to 4 rows of cells.

####### Remarks.

**Figure 1.**
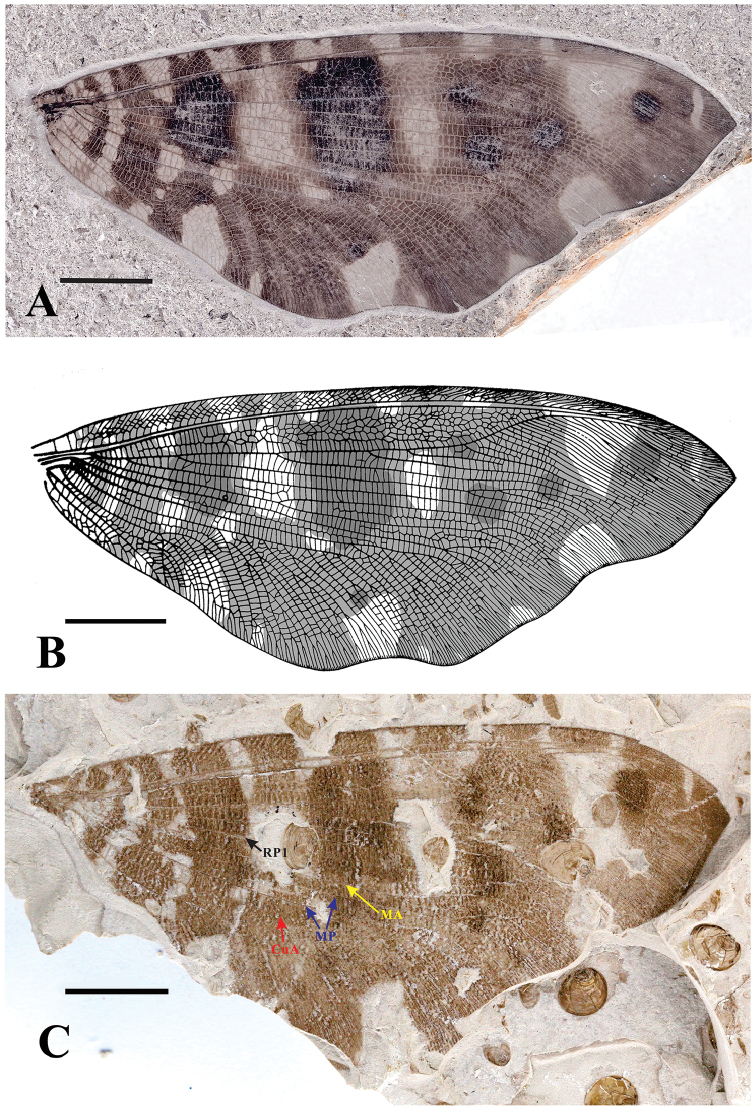
*Laccosmyluscalophlebius* Ren & Yin, 2003. **A** Holotype: CNU-NEU-NN99003, a well-preserved hind wing **B** line drawing of holotype CNU-NEU-NN99003 (modified from Ren and Yin 2003) **C** part of new material CNU-NEU-NN2018006P/C, a well-preserved hind wing. Scale bar: 10 mm.

**Figure 3.**
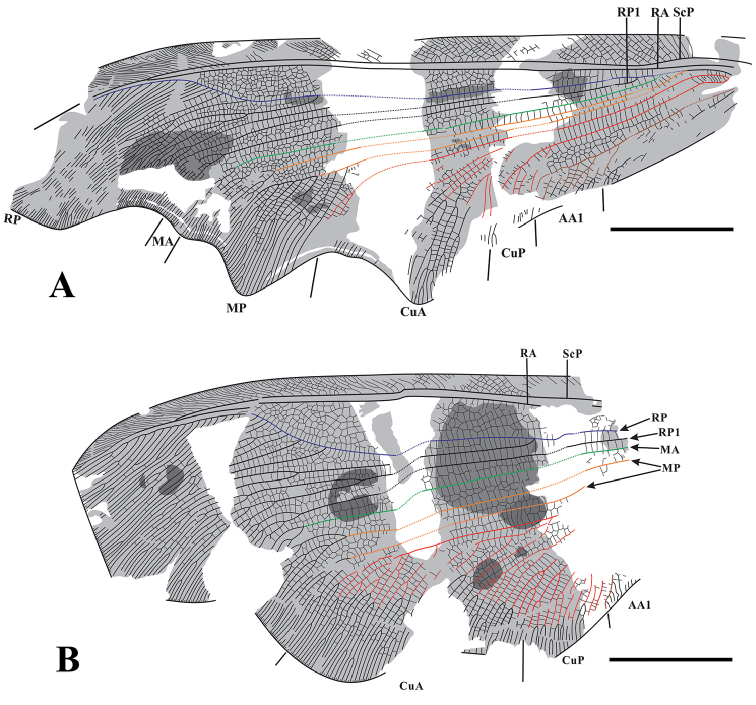
*Laccosmyluscicatricatus* sp. n., Holotype. **A** Line drawing of forewing **B** Line drawing of hind wing. Scale bar: 10 mm.

*Laccosmylus* Ren & Yin, 2003 was assigned to Saucrosmylidae according to its expanded RA area and complicated wing venation of hind wing (Ren & Yin, 2003). Nevertheless, this remarkable genus was established without forewing information which prevent to conduct full comparisons with other saucrosmylids. Herein, the newly collected fossil specimen related to the genus provides significant forewing information to address the issue. The new specimen CNU-NEU-NN2018007P/C of *L.cicatricatus* sp. n. clearly belongs to *Laccosmylus* because of the similar characters of hind wing (Figs 1, 2, 3), i.e. the same broad wing shape with undulant outer margin, the similar patches and spots markings in hind wing, the same expanded RA area with seven rows of irregular cells, the same longitudinal veins and similar cross-veins.

This new species of *L.cicatricatus* sp. n. and *L.latizonus* sp. n. together with a new specimen of *L.calophlebius* Ren & Yin, 2003 provide significant information, especially the supplement of forewing features, to corroborate the status of *Laccosmylus*. *Laccosmylus* is definitely different from *Rudiosmylus* Ren & Yin, 2003, *Ulrikezza* Fang, Ren & Wang, 2015 and *Huiyingosmylus*Liu et al., 2013 by its sharply bent RP at terminal of the wing and unique patch-like and stripe-like wing markings, in contrast to the three genera bearing slightly bent RP and spot-like wing markings (Ren and Yin 2003, Liu et al. 2013, Fang et al. 2015). *Laccosmylus* is different from *Saucrosmylus* Ren & Yin, 2003 by its deeply undulant outer margin of forewing and broad hind wing, while *Saucrosmylus* only has slightly undulant outer margin in both wings and slender hind wing (Ren and Yin 2003). Comparing to *Daohugosmylus*Liu et al., 2014 that was established by an incomplete hind wing, *Laccosmylus* possesses the undulant outer margin of hind wing, pigmented wing marking at the basal part of hind wing and more rows of smaller veinlets between costal veinlets in hind wing, which are absent in *Daohugosmylus* (Liu et al. 2014). Interestingly, *Laccosmylus* possesses similar forewing shape with *Bellinympha*, however, both of the two species of *Bellinympha* have pinnate-like markings on forewings and three continuous markings on hind wings, and bearing slender hind wings (Wang et al. 2010), while *Laccosmylus* develops patch-like or stripe-like wing markings on both wings and bears broad hind wings.

###### Laccosmylus calophlebius

Taxon classificationAnimaliaNeuropteraSaucrosmylidae

Ren & Yin, 2003

Figure 1

####### Emended diagnosis.

Hind wing with many hyaline patch-like markings and mottled with some irregular pigmented patch-like markings, and the costal region with intermittent marking; outer margin undulant. RA-RP area expanded with 6–7 rows of irregular cells. One or two rows of cells present among RP branches and MA-CuA area.

####### Re-description of holotype and description of new material.

Hind wing length approx. 70 mm, width approx. 35 mm. Costal veinlets distally forked, interlinked by 6–7 rows of smaller veinlets. ScP fused with RA apically. RP with 5 main branches before RP sharply bent towards RA anteriorly. MA forked at 1/3 part of hind wing or forked terminally (Figure 1). The first branch of MP forming several dichotomous branches terminally, the second branch of MP with multiple pectinate branches terminally. CuA forming a large triangular area, with numerous oblique pectinate branches. CuP much shorter than CuA, with approx. 8 branches. AA1 and AA2 with several pectinate branches, AA3 short and simple.

####### Material examined.

Holotype: CNU-NEU-NN99003, only hind wing preserved. New material: CNU-NEU-NN2018006P/C, only hind wing preserved. These specimens are deposited in the Key Laboratory of Insect Evolution and Environmental Changes, College of Life Sciences, Capital Normal University, Beijing, China.

####### Type locality and horizon.

Jiulongshan Formation, Daohugou locality (41°18.5'N, 119°13'E (DDM)), Shantou Township, Ningcheng County, Chifeng City, Inner Mongolia, China; Middle Jurassic, Bathonian-Callovian boundary.

####### Remarks.

The new material CNU-NEU-NN2018006P/C evidently belongs to the *L.calophlebius* Ren & Yin, 2003 according to the same hind wing shape, marking and venation. This species was first erected based on only one specimen with a hind wing. Moreover, at that time, this species was the representative with generic diagnosis for the *Laccosmylus*, which led some unilateral diagnosis of the genus and species. Here, the MA in the new specimen shows the previous diagnosis of *L.calophlebius* with MA early branching needs to be changed. In addition, individual differences might have happened frequently in Saucrosmylidae, which has multiple venation.

###### Laccosmylus cicatricatus sp. n.

Taxon classificationAnimaliaNeuropteraSaucrosmylidae

http://zoobank.org/59608202-1E21-4AEA-A975-9863806D7391

Figures 2 , 3

####### Diagnosis.

Large body size, body length more than 37.8 mm, forewing length approx. 60–80 mm, hind wing length approx. 55–76 mm. Forewing distinctly painted with three irregular pigmented marking, outer margin deeply undulant. Hind wing with many hyaline patch-like markings and mottled with some irregular pigmented patch-like markings, and the entire dark costal region. The venation of fore- and hind wings: RA-RP area expanded with 6–7 rows of irregular cells; 1–4 rows of cells present among RP branches and MA-CuA area.

####### Description.

Forewing elongated, with irregularly undulant outer margin. Forewings most heavily pigmented, with two irregular hyaline stripes and a patch-like marking near the outer margin. Trichosors present along distal half of wing margin. Costal veinlets distally forked, interlinked by 6–7 rows of smaller veinlets. ScP fused with RA apically. RA-RP area expanded, with 6–7 rows of irregular cells. RP with five main branches before RP sharply bent towards RA anteriorly. One to three rows of crossveins existing between main longitudinal veins from radius area to anal area. MA forked terminally. The first branch of MP forming several dichotomous branches terminally, the second branch of MP with multiple pectinate branches terminally. CuA forming a large triangular area, with numerous oblique pectinate branches. CuP much shorter and simpler than CuA, with only two main branches. AA1 with approx. eight pectinate branches, while AA2 and AA3 almost invisible.

Hind wing broader and shorter than forewing, with outer margin slightly undulant. The membrane of hind wing covered with numerous dark and hyaline patch-like markings. Trichosors preserved along distal half of wing margin. Venation of hind wing similar to forewing from costal section to media area. CuP in hind wing with approx. eight branches, more complicated than those in forewing. AA1 partially preserved. AA2 and AA3 invisible.

**Figure 2.** *Laccosmyluscicatricatus* sp. n., Holotype: CNU-NEU-NN2018007P/C. **A** part **B** counterpart **C** hind wing of part. Scale bar: 10 mm.

####### Material examined.

Holotype: CNU-NEU-NN2018007P/C, sex unknown, body, and four wings preserved. This specimen is deposited in Inner Mongolia Ningcheng Daohugou Paleontological Protection Museum, Chifeng City, Inner Mongolia, China.

####### Type locality and horizon.

Jiulongshan Formation, Daohugou locality (41°18.5'N, 119°13'E (DDM)), Shantou Township, Ningcheng County, Chifeng City, Inner Mongolia, China; Middle Jurassic, Bathonian-Callovian boundary.

####### Etymology.

The Latin *cicatricatus* is derived from mottles and patches on pigmented wings of this species.

####### Remarks.

The new species belongs to the *Laccosmylus* according to the features of hind wing, i.e. the broad hind wing shape, undulate outer margin, similar venation, and similar distinct colour markings. *L.cicatricatus* sp. n. can be distinguished from the type species *L.calophlebius* by the following characters in hind wing, e.g., the entire dark costal region vs. the costal region with intermittent marking on *L.calophlebius*, and more rows of cells present among RP branches and MA-CuA area than in *L.calophlebius*. In addition, a hyaline spot on the hind wing apex presents in the *L.calophlebius* but absent in *L.cicatricatus* sp. n.

###### Laccosmylus latizonus sp. n.

Taxon classificationAnimaliaNeuropteraSaucrosmylidae

http://zoobank.org/E65B109E-D091-4C8A-B58D-40FB2156CF52

Figures 4 , 5

####### Diagnosis.

Forewing with four pigmented patch-like markings. Hind wing with three pigmented stripes, and the third stripe interlinked with a fuscous patch covering wing apex. The venation of fore- and hind wing: RA area forming 4–5 rows of irregular cells; 1 to 2 rows of cells between longitudinal veins from radius area to anal area.

####### Description.

**Figure 4.**
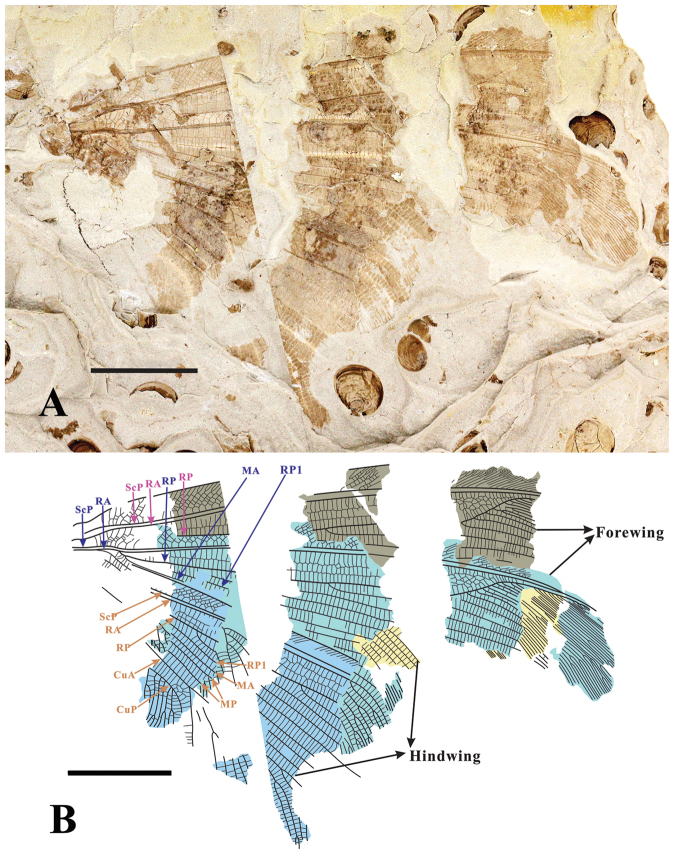
*Laccosmyluslatizonus* sp. n. **A** Paratype: CNU-NEU-NN2018009 with four wings overlapped **B** Line drawing of CNU-NEU-NN2018009. Scale bar: 10 mm.

Forewing more than 54.4 mm long, partially preserved, outer margin unknown (Figure 4A, B). Four pigmented patch-like markings present. Costal veinlets forked twice distally, interlinked by 5–7 rows of smaller veinlets. ScP fused with RA apically. RA-RP area broad, forming 4–5 rows of irregular cells. RP sharply bent towards RA terminally, forming an angle of approx. 45°. Single row of cells between main longitudinal veins from radius area to anal area. MA and MP partially preserved. CuA forming a large triangular area.

**Figure 5.**
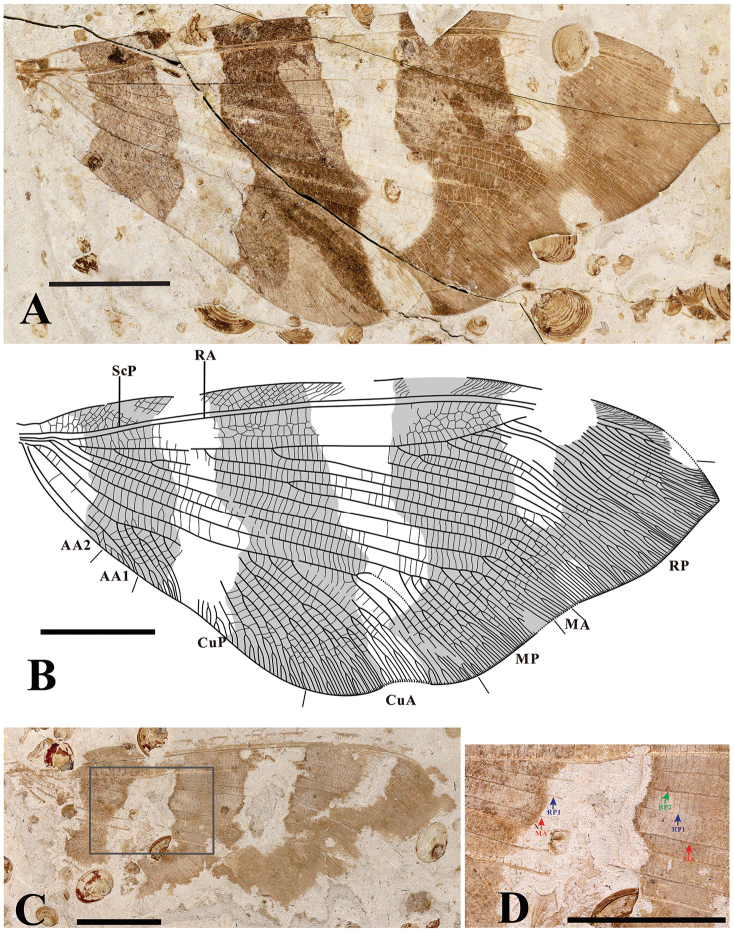
*Laccosmyluslatizonus* sp. n. **A** Part of holotype CNU-NEU-NN2018008P/C, a well-preserved right hind wing **B** Line drawing of hind wing of Holotype **C** Part of paratype CNU-NEU-NN2018010P/C, a well-preserved hind wing **D** Amplification of the rectangle part of C. Scale bar: 10 mm.

Hind wing length 56–59 mm, width 22–26 mm. Hind wing broad, with outer margin slightly undulant, same shape as the type species *L.calophlebius* (Figures 1A, 5A), covered with 3 pigmented stripes, and the third stripe interlinked with a fuscous patch covering wing apex. (Figure 5A, C). Trichosors present along distal half of wing margin. Hind wing showing similar vein pattern as forewing from costal area to media area (Figures 4, 5A, B). In addition, RP1 deeply branched at the base or not (Figure 4A, B, D); MA forming three main branches distally; MP forked before the separation of MA from RP; MP forming several pectinate branches distally; CuP with several pectinate branches. AA1 and AA2 simple, partially preserved.

####### Type material.

**Holotype**: CNU-NEU-NN2018008P/C, only hind wing preserved; **Paratypes**: CNU-NEU-NN2018009, four wings partially preserved and joined together; CNU-NEU-NN2018010P/C, only hind wing partially preserved. These specimens are deposited in the Key Laboratory of Insect Evolution and Environmental Changes, College of Life Sciences, Capital Normal University, Beijing, China.

####### Type locality and horizon.

Jiulongshan Formation, Daohugou locality (41°18.5'N, 119°13'E (DDM)), Shantou Township, Ningcheng County, Chifeng City, Inner Mongolia, China; Middle Jurassic, Bathonian-Callovian boundary.

####### Etymology.

The Latin *latizonus* is derived from the stripe-like wing markings of this species.

####### Remarks.

The new species evidently belongs to *Laccosmylus* according to the emended diagnosis of the genus, i.e., the same broad hind wing shape, undulate outer margin and similar venation, and even the arrangement of wing markings. *Laccosmyluslatizonus* sp. n. can be easily distinguished from *L.calophlebius* and *L.cicatricatus* sp. n. by the following characters of the hind wing markings, i.e. three distinct pigmented stripes in the *L.latizonus* vs. scattered patch-like markings on the other two species. In addition, the venation of these species are also distinctively different, e.g., *L.latizonus* sp. n. with 4–5 rows of irregular cells between RA and RP vs. 6–7 rows of irregular cells in the other two species; relatively simpler cross-veins in *L.latizonus* sp. n. in radial sector than in the other two species.

## Supplementary Material

XML Treatment for Laccosmylus

XML Treatment for Laccosmylus calophlebius

XML Treatment for Laccosmylus cicatricatus

XML Treatment for Laccosmylus latizonus
